# The potential utility of urinary biomarkers for risk prediction in combat casualties: a prospective observational cohort study

**DOI:** 10.1186/s13054-015-0965-y

**Published:** 2015-06-16

**Authors:** Ian J. Stewart, Kristen R. Glass, Jeffrey T. Howard, Benjamin D. Morrow, Jonathan A. Sosnov, Edward D. Siew, Nancy Wickersham, Wayne Latack, Hana K. Kwan, Kelly D. Heegard, Christina Diaz, Aaron T. Henderson, Kristin K. Saenz, T. Alp Ikizler, Kevin K. Chung

**Affiliations:** San Antonio Military Medical Center, 3551 Roger Brooke Drive, JBSA Ft Sam, Houston, TX 78234-6200 USA; Uniformed Services University of the Health Sciences, 4301 Jones Bridge Rd, Bethesda, MD 20814 USA; United States Army Institute of Surgical Research, 3698 Chambers Pass STE B, JBSA Ft Sam, Houston, TX 78234-7767 USA; Vanderbilt University Medical Center, 1211 Medical Center Drive, Nashville, TN 37232 USA; Kessler Medical Center, 301 Fisher St, Keesler AFB, MS 39534 USA; Eglin Hospital, 307 Boatner Road, Eglin Air Force Base, FL 32542 USA; David Grant Medical Center, 101 Boden Circle, Travis Air Force Base, CA 94535 USA

## Abstract

**Introduction:**

Traditional risk scoring prediction models for trauma use either anatomically based estimations of injury or presenting vital signs. Markers of organ dysfunction may provide additional prognostic capability to these models. The objective of this study was to evaluate if urinary biomarkers are associated with poor outcomes, including death and the need for renal replacement therapy.

**Methods:**

We conducted a prospective, observational study in United States Military personnel with traumatic injury admitted to the intensive care unit at a combat support hospital in Afghanistan.

**Results:**

Eighty nine patients with urine samples drawn at admission to the intensive care unit were studied. Twelve patients subsequently died or needed renal replacement therapy. Median admission levels of urinary cystatin C (CyC), interleukin 18 (IL-18), L-type fatty acid binding protein (LFABP) and neutrophil gelatinase-associated lipocalin (NGAL) were significantly higher in patients that developed the combined outcome of death or need for renal replacement therapy. Median admission levels of kidney injury molecule-1 were not associated with the combined outcome. The area under the receiver operating characteristic curves for the combined outcome were 0.815, 0.682, 0.842 and 0.820 for CyC, IL-18, LFABP and NGAL, respectively. Multivariable regression adjusted for injury severity score, revealed CyC (OR 1.97, 95 % confidence interval 1.26-3.10, p = 0.003), LFABP (OR 1.92, 95 % confidence interval 1.24-2.99, p = 0.004) and NGAL (OR 1.80, 95 % confidence interval 1.21-2.66, p = 0.004) to be significantly associated with the composite outcome.

**Conclusions:**

Urinary biomarker levels at the time of admission are associated with death or need for renal replacement therapy. Larger multicenter studies will be required to determine how urinary biomarkers can best be used in future prediction models.

**Electronic supplementary material:**

The online version of this article (doi:10.1186/s13054-015-0965-y) contains supplementary material, which is available to authorized users.

## Introduction

A variety of risk prediction scores, including the injury severity score (ISS) [1] and the revised trauma score (RTS) [2], can be used to predict mortality after traumatic injury. These models are based on anatomical injury descriptions (in the case of ISS) and physiologic parameters to include vital signs and the Glasgow coma scale (in the case of the RTS). Functional markers of organ dysfunction, such as urinary biomarkers (UBs), may provide additional information that could be used to refine these models.

In the care of combat injured patients, risk prediction is especially important as military physicians have limited resources and triage decisions must be rapid and accurate. The ability to identify patients who will require a large amount of resources is paramount, but is a process currently based on clinical intuition. An objective test is needed to support the decision making process. Furthermore, studies in combat injured patients represent a homogeneous population of young, previously healthy subjects. This might provide insights into subpopulations of civilian trauma populations and aid in the refinement of risk prediction models.

Given the paucity of data on UBs in the setting of trauma, we sought to determine the potential utility of UBs in an austere deployed environment to predict poor outcomes. We hypothesized that UBs would predict a combined outcome of severe acute kidney injury (AKI), defined by the need for renal replacement therapy (RRT), or death.

## Methods

The study took place at Craig Joint Theater Hospital (CJTH) in Bagram Airfield, Afghanistan from October 2012 to December 2013. We included US military personnel with traumatic injury that required ICU-level care. Patients were excluded if they did not have a Foley catheter or if more than 48 hours had elapsed since injury. The study was approved by the institutional review board responsible for studies performed in theater (the US Army Medical Research and Materiel Command). Informed consent was deemed necessary if the subject was able to provide consent. However, given the inability to access legally authorized representatives, this requirement was waived given the low risk nature of the study for patients unable to consent.

We collected urine samples within 3 hours of admission to the ICU. Within 1 hour, samples were centrifuged at 2,000 g for 10 minutes and then frozen at −80 °C. Samples were batched and shipped back to the United States using a commercial carrier. A temperature logger was included in the sample shipment to ensure that the samples remained at an appropriate temperature (less than −40 °C). Urinary biomarkers measured included neutrophil gelatinase-associated lipocalin (NGAL), cystatin C (CyC), kidney injury molecule-1 (KIM-1), interleukin (IL)-18, and L-type fatty acid-binding protein (L-FABP). Results were obtained using commercially available kits for NGAL (RnD Systems, Minneapolis, MN, USA), KIM-1 (RnD Systems), IL-18 (MBL International, Woburn, MA, USA), L-FABP (Hycult Biotech, Plymouth Meeting, PA, USA), and CyC (RnD Systems). All samples were run twice and the average of these two values was used for analysis. The results were normalized for urine concentration by dividing by the urinary creatinine concentration.

Data collection was accomplished in both a prospective and retrospective fashion. Data on demographics, ISS [1], blood transfusions and laboratory values were collected prospectively by the investigators at CJTH. Massive transfusion was defined at ≥10 units of packed red blood cells in a 24 hour period. Data on events that occurred after patient evacuation from CJTH, to include the need for RRT at Landstuhl Regional Medical Center (a military hospital in Germany, which is the next step in the evacuation chain) and in-hospital death were collected retrospectively. Specific criteria to start RRT were not part of the protocol. Physicians who were not involved in the study determined if the patients needed RRT. Patients who died at any point during their initial hospitalization were considered as meeting the mortality component of the composite outcome.

The combined outcome was defined as a composite of death or the need for RRT. To examine the differences in UB concentrations between those that developed the combined outcome and those that did not we utilized the Wilcoxon rank sum test. To determine the diagnostic characteristics, we generated receiver operator characteristic (ROC) curves and evaluated the area under the curves (AUC). We then determined sensitivity, specificity, positive predictive value and negative predictive value at the point of optimal test performance by determining the probability cutoff values in which (1) the overall percent of cases correctly classified > = 70 %, and (2) sensitivity and specificity both exceeded 60 %, or the cutoffs as close to these criteria as possible [3]. These criteria were used as a way to balance the tradeoff between sensitivity, specificity and overall prediction accuracy inherent in the determination of predictive cutoff values. To see if UBs provided additional information to that which would possibly be clinically available, we performed logistic regression models with independent variables of ISS and UBs (normalized for creatinine concentration) with the dependent variable of the composite outcome. Separate models were run for each of the five UBs. A two-tailed *p* value <0.05 was considered significant. Statistics were performed using SAS version 9.2 (Cary, NC, USA).

## Results

**Fig. 1:**
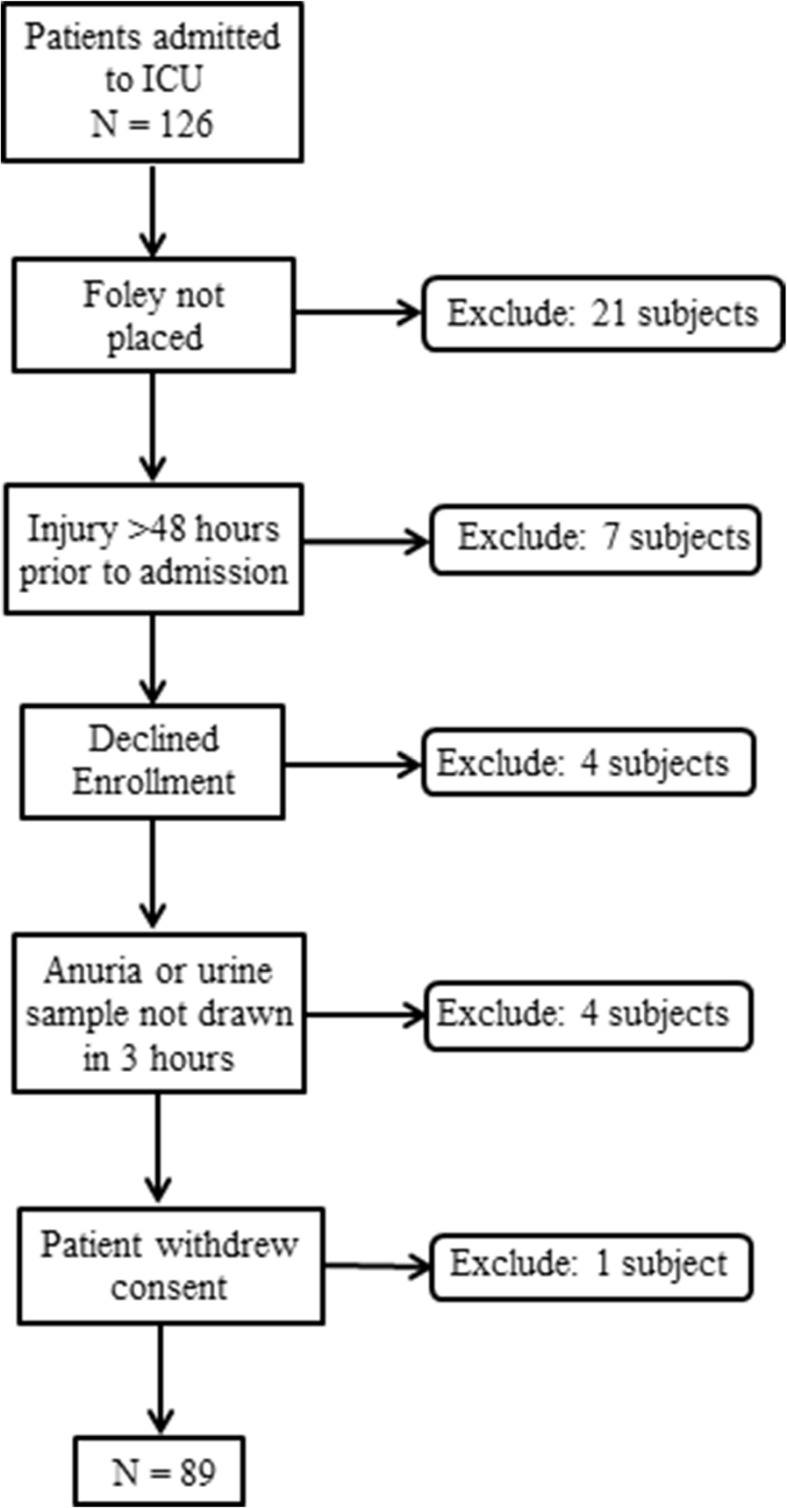
Cohort derivation

Over the course of the study, 126 subjects were admitted to the ICU at CJTH. Of these, 37 patients were excluded leaving 89 subjects for analysis. As noted in Fig. 1, patients were excluded for not having a Foley catheter (21 patients), injury more than 48 hours before admission (7 patients), declined enrollment (4 patients), anuria (2 patients), urine sample not collected within 3 hours (2 patients) or withdrawal of consent (1 patient).

The baseline characteristics of study subjects are presented in Table 1. Average age was 26.9 ± 5.3 years and the majority (96.6 %) was male. Median (IQR) ISS was 18 (11, 38) denoting severe injury. Median lactate at admission was 1.8 (1.1, 2.9) mmol/L and 37.1 % of patients required a massive transfusion. Patients who developed the composite endpoint were more severely injured as evidenced by significantly higher ISS and lactates. More patients who developed the composite endpoint required a mass transfusion. Six patients (6.7 %) required RRT. The start of RRT occurred at a median of 3 days after injury (range 1–8 days). Nine patients (10.1 %) died. Median hospital day of death was 4 (3, 13) and ranged from 2 to 38. Of the six patients who required RRT, three (50 %) died. Urine samples were drawn at an average of 19.7 ± 10.8 hours after injury. At the time that urine samples were drawn, 18.0 % of patients (n = 16) had AKI by the Kidney Disease Improving Global Outcomes (KDIGO) [4] criteria. The majority of patients (14.6 %) were KDIGO stage 1 with 2.3 % and 1.1 % having KDIGO stages 2 and 3, respectively. A further 17 patients developed AKI after urine sampling, resulting in an overall rate of AKI of 37.1 %. KDIGO stages 1 through 3 occurred in 25.8 %, 4.5 % and 6.7 % of patients, respectively. The overall rates of AKI in this cohort have previously been described [5]. This should be understood in the context of the limitations of the currently used standard for the diagnosis of AKI, serum creatinine, given that resuscitation may affect these classifications [6].

**Table 1:** Baseline characteristics of the patient cohort

Characteristic	Full cohort	Without combined endpoint*	Combined endpoint*	*P* value
Number	89	77	12	-
Age, years	26.9 ± 5.3	26.8 ± 5.4	27.8 ± 4.9	0.57
Male, %	96.6	96.1	100	1.0
Median ISS (IQR)	18 (11, 38)	18 (9, 34)	46 (28.5, 67)	0.001
Median lactate (IQR)	1.8 (1.1, 2.9)	1.5 (1.1,2.5)	4.3 (2.1-6.7)	0.001
Mass transfusion, %	37.1	31.2	75.0	0.008
RRT, %	6.7	0	50	-
Mortality, %	10.1	0	75.0	-
Combined endpoint, %	13.5	-	-	-

Data are expressed as mean ± SD unless stated otherwise. *Combined endpoint is need for RRT or death. *ISS* injury severity score, *RRT* renal replacement therapy

**Fig. 2:**
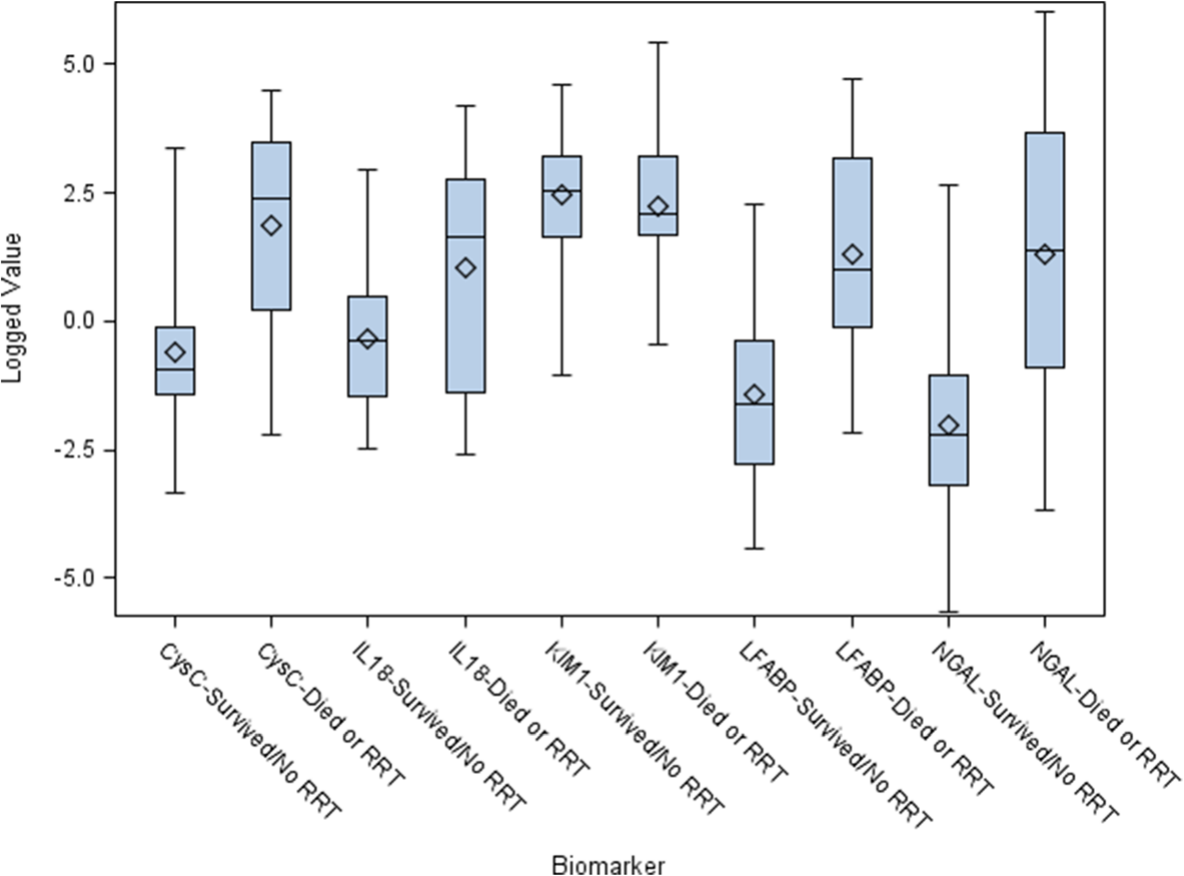
Distribution of log-transformed urinary biomarker values stratified by the presence or absence of death or renal replacement therapy. *Boxes* represent the median (*middle line*) and interquartile range and *diamonds* represent the mean. The *error bars* indicate the maximum and minimum values. *CyC* cystatin C, *IL-18* interleukin-18, *KIM-1*: kidney injury molecule-1, *L-FABP* liver-type fatty acid-binding protein, *NGAL* neutrophil gelatinase-associated lipocalin

The median (IQR) of cystatin C (CyC) for those who developed versus those who did not develop the outcome were 12.95 ng/mg creatinine (1.24, 32.61) and 0.38 ng/mg creatinine (0.24, 0.87), respectively (*p* <0.001). Median (IQR) of IL-18 was 5.34 pg/mg creatinine (0.26, 17.50) for patients with the primary outcome and 0.69 pg/mg creatinine (0.23, 1.64) for those without (*p* = 0.04). For KIM-1, there was no difference between those with and without the outcome, 8.19 pg/mg creatinine (5.38, 27.26) and 12.84 pg/mg creatinine (5.22, 25.29), respectively (*p* = 0.59). The median values for LFABP were higher in those who developed the composite outcome, 3.35 ng/mg creatinine (0.89, 25.06), versus 0.20 ng/mg creatinine (0.06, 0.69), *p* <0.001. Finally, NGAL was also significantly higher in those who had the outcome compared to those that did not, 4.05 ng/m creatinine g (0.41, 41.06) and 0.11 ng/mg creatinine (0.04, 0.34), respectively, *p* <0.001. There were no significant differences noted between patients who required RRT by the end of the third hospital day compared to patients who required RRT later in their hospital course. Figure 2 demonstrates the log-transformed values of the UBs stratified by the presence or absence of the combined outcome. Additional file 1 demonstrates the non-log-transformed UB to creatinine ratios.

**Fig. 3:**
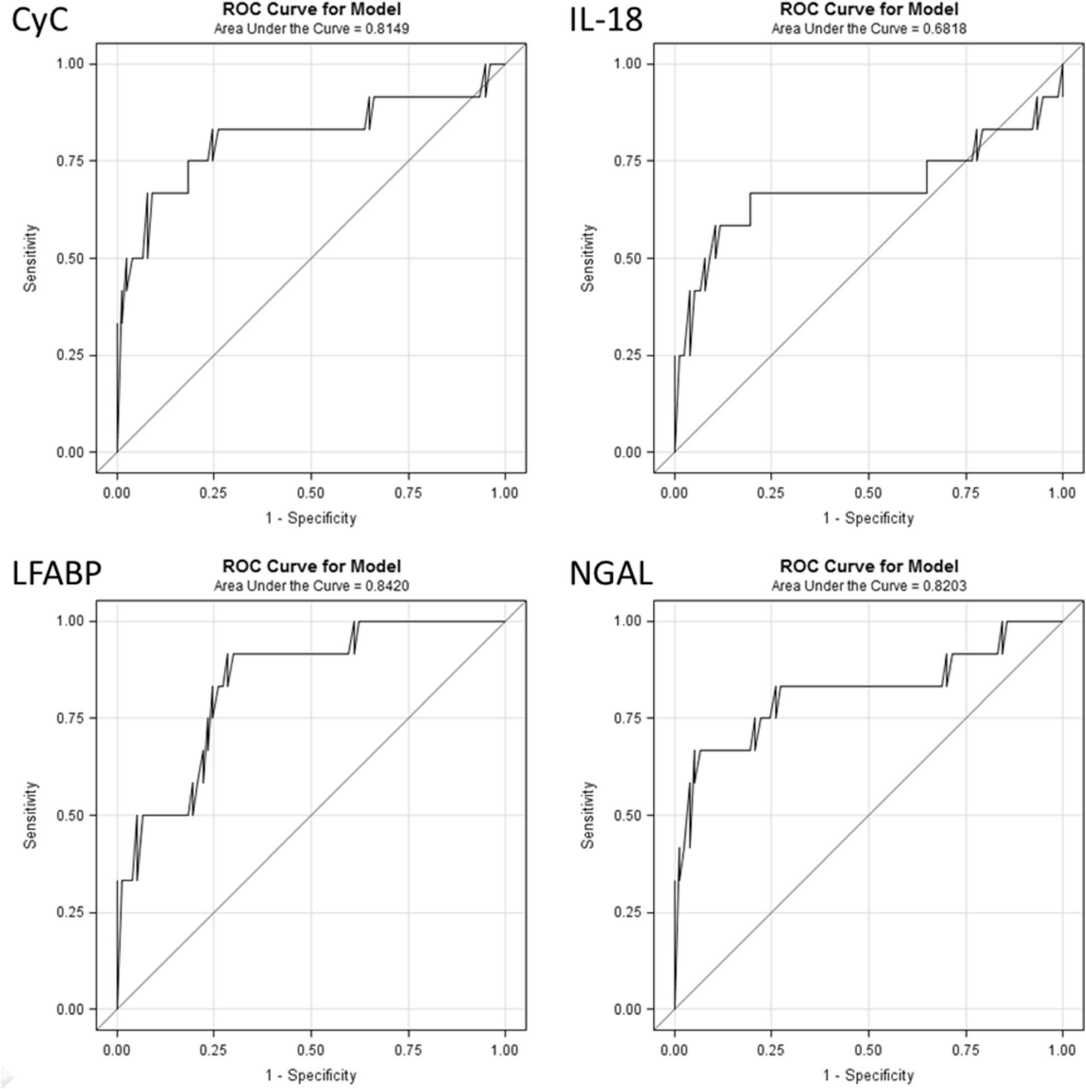
Receiver operator characteristics curve for urinary biomarkers for the combined outcome. *ROC* receiver operator characteristic, *CyC* cystatin C, *LFABP* L-type fatty acid-binding protein, *NGAL* neutrophil gelatinase-associated lipocalin

We examined the relations between the UB to creatinine ratios and the composite endpoint in logistic regression models by examining both the UB to creatinine ratio (Additional file 2) and the log of the UB to creatinine ratio (Table 2). While both methods arrive at the same AUCs (given inherent rank ordering), the logged values better described the relationship. The ROCs for each of the biomarkers that were statistically significant are presented in Fig. 3. The AUCs for CyC, IL-18, LFABP and NGAL were 0.815, 0.682, 0.842 and 0.820, respectively. Table 2 shows both the unadjusted and ISS adjusted logistic regression models. After adjustment, Cyc C (odds ratio (OR) 1.97, 95 % CI 1.26, 3.10; *p* = 0.003), LFABP (OR 1.92, 95 % CI 1.24, 2.99; *p* = 0.004) and NGAL (OR 1.80, 95 % CI 1.21, 2.66; *p* = 0.004) remained significant. IL-18 (OR 1.45, 95 % CI 0.96, 2.20; *p* = 0.08) was not significant after adjustment. Additional file 3 reports these same models using the log mean biomarker levels not corrected for creatinine. These results are similar with two exceptions. First, IL-18 was not significant in the univariate model. Second, in the multivariate model, increasing KIM-1 concentrations were associated with a decrease in the combined endpoint. We also examined more traditional markers of AKI, including creatinine and the average of the first 2 hours of urine output. Both creatinine (OR 55.99, 95 % CI 4.59, 682.57; *p* = 0.002) and urine output (OR 1.04, 95 % CI 1.01, 1.07; *p* = 0.02) were significant for the composite outcome. While creatinine had a very high odds ratio, it is important to note the wide confidence intervals. Therefore this result should be interpreted with caution.

**Table 2:** Univariate and injury severity score-adjusted models for the combined outcome

Marker	Univariate			Adjusted for ISS	
	OR (95 % CI)	*P* value	AUC	OR (95 % CI)	*P* value
CyC	2.19 (1.48, 3.26)	<0.001	0.815	1.97 (1.26, 3.10)	0.003
IL-18	1.68 (1.13, 2.49)	0.01	0.682	1.45 (0.96, 2.20)	0.08
KIM-1	0.85 (0.50, 1.44)	0.54	0.549	0.61 (0.31, 1.18)	0.14
LFABP	2.13 (1.41, 3.22)	<0.001	0.842	1.92 (1.24, 2.99)	0.004
NGAL	1.96 (1.37, 2.81)	<0.001	0.820	1.80 (1.21, 2.66)	0.004
ISS	1.05 (1.02, 1.08)	0.002	0.790	-	-

Odds ratio (*OR*) represents per one log increase in biomarker-to-creatinine ratio and one unit increase in injury severity score. *CyC* cystatin C, *IL-18* interleukin-18, *KIM-1*, kidney injury molecule-1, *L-FABP* liver-type fatty acid-binding protein, *NGAL* neutrophil gelatinase-associated lipocalin, ISS, injury severity score

The pre-specified criteria for cutoff were satisfied by all biomarkers except KIM-1, which met criteria for sensitivity at a cutoff of 0.115. All other possible cutoff values for KIM-1 failed to meet any of the three criteria above. Optimal discriminative values for CyC, IL-18, KIM-1, LFABP, NGAL and ISS were 2.24 ng/mg creatinine, 2.21 pg/mg creatinine, 31.1 pg/mg creatinine, 1.76 ng/mg creatinine, 1.51 ng/mg creatinine, and 27, respectively. The sensitivity, specificity, positive predictive value and negative predictive values at these cutoffs are presented in Table 3. With the exception of KIM-1, UBs demonstrated reasonable sensitivity (0.5 − 0.67) and specificity (0.79 − 0.91) for predicting the combined outcome.

**Table 3:** Sensitivity, specificity, negative predictive value and positive predictive value at optimal test characteristics

Marker	Optimal discriminant value*	Sensitivity	Specificity	Positive predictive value	Negative predictive value
CyC	2.24 ng/mg	0.67	0.84	40 %	94 %
IL-18	2.21 pg/mg	0.67	0.79	33 %	94 %
KIM-1	31.1 pg/mg	0.75	0.14	12 %	78 %
LFABP	1.76 ng/mg	0.50	0.83	32 %	91 %
NGAL	1.51 ng/mg	0.67	0.91	53 %	95 %
ISS	27	0.75	0.65	25 %	94 %

*Units are expressed in ng/mg creatinine or pg/mg creatinine. *CyC* cystatin C, *IL-18* interleukin-18, *KIM-1* kidney injury molecule-1, *L-FABP* liver-type fatty acid-binding protein, *NGAL* neutrophil gelatinase-associated lipocalin, *ISS* injury severity score

## Discussion

We found that UBs drawn at admission predicted the subsequent need for RRT, and death. In the case of CyC, LFABP and NGAL, the relationship remained significant after adjustment for injury severity. As a stand-alone test, NGAL with a cutoff of 1.51 ng/mg creatinine had a positive predictive value of 53 % and a negative predictive value of 95 %. This information also suggests that UBs might improve the predictive capability of commonly used trauma scoring systems. In the military setting, this information could be used to triage patients and assign prioritization for aeromedical evacuation.

Prior work has also shown the ability of UBs to predict both mortality and the need for RRT. Urinary NGAL is perhaps the best studied. A pooled meta-analysis in 2009 that examined the diagnostic characteristics of urinary NGAL for the outcomes of RRT and death found AUCs of 0.782 and 0.706, respectively. Since the publication of this analysis, further evidence has been published supporting the association of NGAL with subsequent need for RRT or with death. In a mixed ICU cohort of 529 patients, NGAL was found to have sensitivity, specificity, positive predictive value and negative predictive value of 0.47, 0.78, 0.20 and 0.94, respectively for the outcome of mortality in 7 days [7]. Test characteristics for the outcome of RRT within 7 days were 0.64, 0.76, 0.07 and 0.99, respectively [7]. Another large ICU cohort of 632 patients found that NGAL had AUCs of 0.89 and 0.64 for the outcome of RRT and mortality, respectively. These numbers are broadly comparable to what we found in our smaller cohort, implying that the information is generalizable. Further evidence also supports the association between elevated NGAL levels and the subsequent need for RRT, or death [8, 9]. Importantly, in one ICU cohort, the association between NGAL levels and the combined endpoint was significant after adjustment for age, acute physiology and chronic health evaluation (APACHE) II scores, presence of organ failure, serum creatinine, sepsis and ICU location [9]. Similarly, in patients undergoing cardiac surgery, a dose response between quintiles of urinary NGAL and the need for RRT was observed, again after adjustment for patient factors [10]. An association of other biomarkers, including IL-18 [7, 9, 10] and CyC [7, 11, 12] with death and RRT has also been observed. The evidence for KIM-1 is conflicting, with one study demonstrating an association with death and RRT [13] and another study failing to demonstrate an association [7]. As KIM-1 appear to peak later than other UBs (approximately 48 hours [14]), it is possible that differences in timing of urine sampling explains this variation. Notably, we only included patients who were admitted within 48 hours of injury, which might explain why we failed to observe an association between KIM-1 and the combined outcome.

There is evidence that elevated UBs even in the absence of overt AKI [15, 16] or after adjustment for AKI [9] increases the risk of mortality. This association is biologically plausible by two possible mechanisms. First, the kidneys receive approximately 20 % of cardiac output [17] and are sensitive to ischemic injury. Ischemia in the kidney, reflected by an increase of UBs, may therefore be indicative of global ischemia. Another possibility is that AKI itself may contribute to death. Post trauma, AKI has been demonstrated to significantly modulate pro- and anti-inflammatory cytokines [18]. These changes in the immune system are thought to be vital in organ-organ interactions, which may result in failure of other organs [19]. Retrospective work in the trauma population showing that AKI is a stronger predictor of multi-organ failure and death than hepatic, cardiac or pulmonary dysfunction confirms this possibility [20].

During combat operations in Iraq and Afghanistan over the past decade, rapid evacuation of injured patients out of theater, where RRT was not routinely unavailable, was essential for the care of injured service members with life-threatening AKI [21]. However, in the future rapid evacuation may be hampered by geographical challenges or prolonged transport times from point of injury to definitive care facilities. The ability to identify patients at high risk, in order to prioritize evacuation, is therefore highly desirable. Our work shows that UBs can identify patients with combat injury who are at high risk of requiring RRT or dying. The high negative predictive value, albeit at some expense to the positive predictive value, is of particular clinical significance to military physicians. In a combat setting, it would be preferable to evacuate a casualty who did not subsequently develop a significant complication than to not evacuate one that did, because the latter would require a large amount of resources, which are limited commodities in the deployed setting. A strategy that involves UB measurement at admission and triage to rapid evacuation could save vital resources, conserving supplies and enabling military physicians to care for a larger number of casualties.

This work might also aid in the further development of mortality risk scores in the civilian setting. Commonly used scores for risk prediction after trauma include ISS and the RTS [2]. Our work suggests that the addition of UBs to these models may improve adverse event prediction, including mortality and the need for RRT. There is limited evidence for the potential role of UBs for the prediction of mortality in patients with burns or traumatic injury. A study of 31 trauma patients admitted to an ICU found correlation with NGAL levels and subsequent AKI, but did not examine the outcomes of mortality or need for RRT [22]. In another study of 90 patients with burn injury (with total body surface area burn >20 %), urinary NGAL obtained shortly after admission was found to be associated with both early (<3 days) and late (>3 days) mortality [23]. Of note, elevated NGAL predicted mortality even after adjustment for age, TBSA, inhalation injury and serum creatinine. Taken in the context of our study, UBs drawn at admission may improve risk prediction models in trauma patients. Future work will be required to examine this hypothesis. A priori stratification by age and co-morbid conditions should be considered for future trials.

Our study has several limitations. We had initially sought to enroll 226 subjects; however, the study took place during a drawdown of combat forces in Afghanistan which decreased enrollment. The smaller number (89) limits the statistical power of the study and necessitated the use of a combined outcome based on both death and the need for RRT. Second, it was not possible to prospectively collect all of the data and some variables had to be examined retrospectively. Last, we do not have data on long-term outcomes for these patients. Indeed, there are a paucity of long-term follow up data on patients with combat injury. However, UBs may have the ability to predict long-term mortality [24]. Future studies should examine the hypothesis that UBs predict long-term outcomes independent of injury severity. These limitations should be considered in the context in which the study was conducted - a resource-poor combat environment. Indeed, in the history of the wars in Iraq and Afghanistan, this is the only work to successfully obtain urine samples for analysis.

## Conclusions

In conclusion, UBs are associated with the combined outcome of death or need for RRT in combat casualties. While this work is preliminary, UBs may have a role for risk prediction in the setting of traumatic injury. Larger multicenter studies will be required to determine how UBs can best be used in future prediction models.

## Key messages

- Urinary biomarker levels drawn at ICU admission predict a combined endpoint of death or renal replacement therapy in combat casualties.
- The relationship between the combined endpoint and three of the urinary biomarkers studied, cystatin C, L-type fatty acid-binding protein and neutrophil gelatinase-associated lipocalin, remained significant even after adjustment for injury severity.
- Urinary biomarkers may provide additional information to existing trauma severity scores to improve risk prediction.
- In a combat or otherwise limited resource setting, this information could be used to prioritize patients for evacuation.

## Additional files

Additional file 1:
**Figure shows distribution of non-log-transformed urinary biomarker to creatinine ratios stratified by the presence or absence of death or renal replacement therapy.**
Additional file 2:
**Table shows univariate and injury severity score-adjusted models for the combined outcome with urinary biomarker levels corrected for creatinine.**
Additional file 3:
**Table shows univariate and injury severity score-adjusted models for the combined outcome with urinary biomarker levels not corrected for creatinine.**

## Abbreviations

AKI: acute kidney injury
AUC: area under the curve
CJTH: Craig Joint Theater Hospital
CyC: cystatin C
IL: interleukin
IQR: interquartile range
ISS: injury severity score
KDIGO: Kidney Disease Improving Global Outcomes
KIM-1: kidney injury molecule-1
L-FABP: L-type fatty acid-binding protein
NGAL: neutrophil gelatinase-associated lipocalin
OR: odds ratio
ROC: receiver operator characteristic curve
RRT: renal replacement therapy
RTS: revised trauma score
UB: urinary biomarker
