# Supplementary material for: The potential utility of urinary biomarkers for risk prediction in combat casualties: a prospective observational cohort study
Source: Crit Care. 2015 Jun 16;19(1):252. doi: 10.1186/s13054-015-0965-y (PMC4487799; doi:10.1186/s13054-015-0965-y)
Supplement: Additional file 1: — Figure shows distribution of non-log-transformed urinary biomarker to creatinine ratios stratified by the presence or absence of death or renal replacement therapy. [file 13054_2015_965_MOESM1_ESM.pdf]

Distribution of non-log$_{10}$ transformed urinary biomarker to creatinine ratios stratified by the presence or absence of death or renal replacement therapy

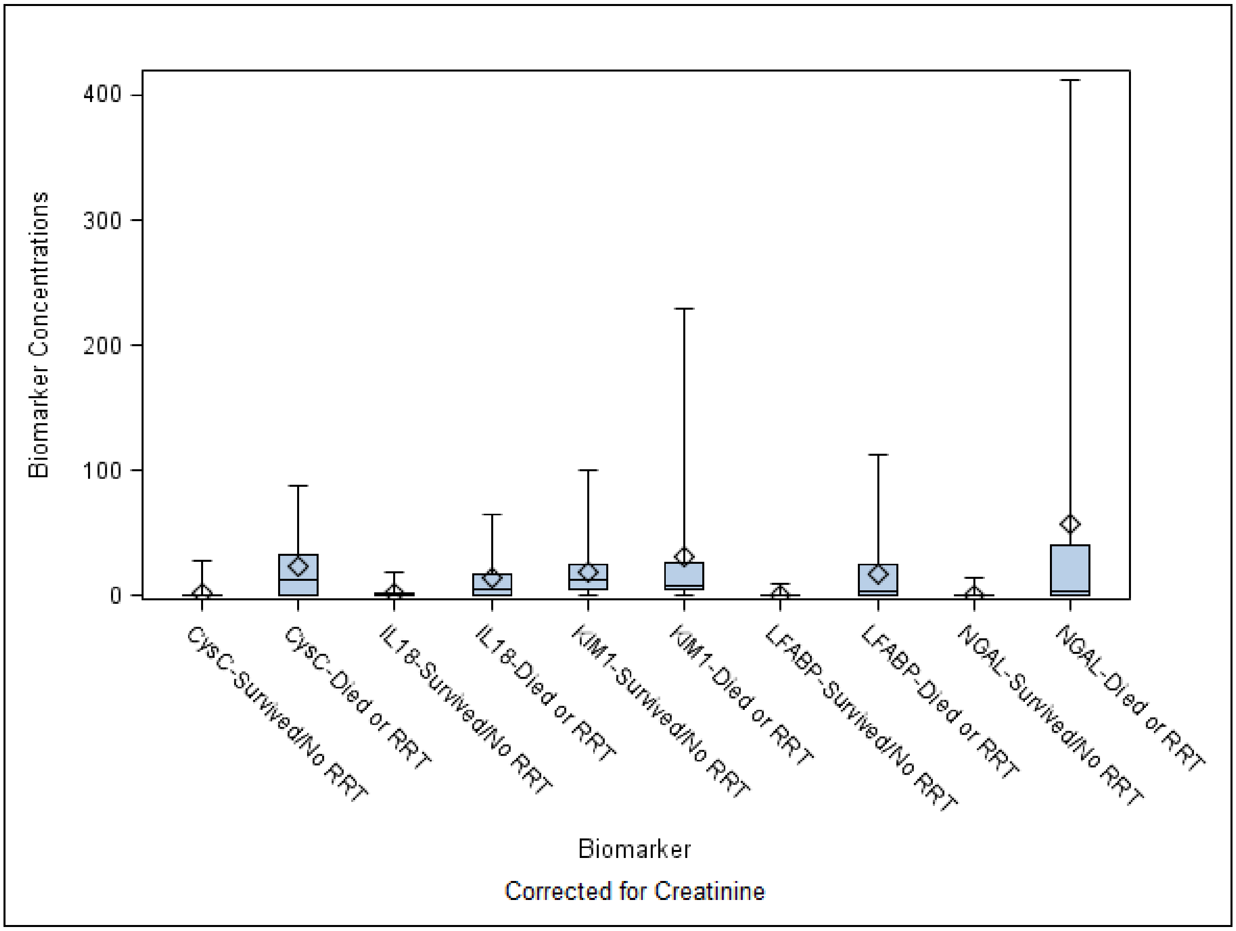

Boxes represent the median (middle line) and interquartile range and diamonds represent the mean. The error bars indicate the maximum and minimum values.

CyC: cystatin C

IL-18: interleukin-18

KIM-1: kidney injury molecule-1

L-FABP: liver-type fatty acid-binding protein

NGAL: neutrophil gelatinase-associated lipocalin
