# Supplementary material for: The potential utility of urinary biomarkers for risk prediction in combat casualties: a prospective observational cohort study
Source: Crit Care. 2015 Jun 16;19(1):252. doi: 10.1186/s13054-015-0965-y (PMC4487799; doi:10.1186/s13054-015-0965-y)
Supplement: Additional file 2: — Table shows univariate and injury severity score-adjusted models for the combined outcome with urinary biomarker levels corrected for creatinine. [file 13054_2015_965_MOESM2_ESM.pdf]

Univariate and injury severity score adjusted models for the combined outcome with urinary biomarker levels corrected for creatinine

Marker*	Univariate			Adjusted for ISS	
	OR (95% CI)	P value	AUC	OR (95% CI)	P value
**CyC**	1.13 (1.05-1.22)	0.001	0.815	1.11 (1.02-1.21)	0.01
**IL-18**	1.15 (1.03-1.28)	0.01	0.682	1.14 (1.02-1.27)	0.03
**LFABP**	1.26 (1.04-1.53)	0.02	0.842	1.20 (0.99-1.47)	0.07
**NGAL**	1.28 (1.02-1.62)	0.04	0.820	1.19 (0.95-1.49)	0.13
**ISS**	1.05 (1.02-1.08)	0.002	0.790	-	-

OR: Odds ratio, these represent per one unit increase in biomarker:creatinine ratio and one unit increase in injury severity score
CI: Confidence interval
CyC: cystatin C
IL-18: interleukin-18
KIM-1: kidney injury molecule-1
L-FABP: liver-type fatty acid-binding protein
NGAL: neutrophil gelatinase-associated lipocalin
ISS: Injury severity score
*Note that KIM-1 could not be modeled as a continuous variable
