# Supplementary material for: The potential utility of urinary biomarkers for risk prediction in combat casualties: a prospective observational cohort study
Source: Crit Care. 2015 Jun 16;19(1):252. doi: 10.1186/s13054-015-0965-y (PMC4487799; doi:10.1186/s13054-015-0965-y)
Supplement: Additional file 3: — Table shows univariate and injury severity score-adjusted models for the combined outcome with urinary biomarker levels not corrected for creatinine. [file 13054_2015_965_MOESM3_ESM.pdf]

**Univariate and injury severity score adjusted models for the combined outcome with urinary biomarker levels not corrected for creatinine**

| Marker | Univariate | | | Adjusted for ISS | |
|---|---|---|---|---|---|
| | OR (95% CI) | P value | AUC | OR (95% CI) | P value |
| **CyC** | 2.02 (1.35-3.03) | <0.001 | 0.810 | 1.73 (1.11-2.72) | 0.02 |
| **IL-18** | 1.46 (0.98-2.18) | 0.07 | 0.653 | 1.27 (0.82-1.96) | 0.29 |
| **KIM-1** | 0.70 (0.44-1.12) | 0.13 | 0.602 | 0.54 (0.30-0.97) | 0.04 |
| **LFABP** | 2.06 (1.32-3.22) | 0.002 | 0.797 | 1.79 (1.11-2.89) | 0.02 |
| **NGAL** | 1.93 (1.34-2.78) | <0.001 | 0.817 | 1.73 (1.16-2.57) | 0.007 |
| **ISS** | 1.05 (1.02-1.08) | 0.002 | 0.790 | - | - |

OR: Odds ratio, these represent per one $\log_{10}$ increase in biomarker concentration and one unit increase in injury severity score
CI: Confidence interval
CyC: cystatin C
IL-18: interleukin-18
KIM-1: kidney injury molecule-1
L-FABP: liver-type fatty acid-binding protein
NGAL: neutrophil gelatinase-associated lipocalin
ISS: Injury severity score
